# Vasospasm following aneurysmal subarachnoid hemorrhage: prediction, detection, and intervention

**DOI:** 10.1186/s41983-018-0050-y

**Published:** 2019-01-08

**Authors:** Hassan Gamal Eldeen Nassar, Azza Abbas Ghali, Wafik Said Bahnasy, Mostafa Mohamed Elawady

**Affiliations:** Department of Neuropsychiatry, Faculty of Medicine, Tanta University, 31527 Tanta, Egypt

**Keywords:** Aneurysmal subarachnoid hemorrhage, Transcranial duplex, EEG monitoring, Angioplasty, Intra-arterial nimodipine

## Abstract

**Background:**

Vasospasm of the cerebral blood vessels is a common complication of aneurysmal subarachnoid hemorrhage (aSAH) which results in delayed cerebral ischemia (DCI) and worsening of the outcome.

**Methods:**

This study was performed on 41 aSAH patients diagnosed by non-contrast brain CT, CT angiography, and digital subtraction angiography followed by interventional aneurysmal embolization. Patients were followed up for 20 days by clinical assessment, EEG monitoring, and transcranial duplex studies (TCD) for early detection of vasospasm and DCI.

**Results:**

The most common ruptured aneurysmal sites were middle cerebral, anterior communicating, posterior communicating, terminal internal carotid, and anterior cerebral arteries respectively. The incidence of vasospasm was 36.8% of the included cases; 57% progressed to DCI while 43% passed a spontaneous regressive course. The most common arteries undergoing vasospasm were the MCA followed by the ACA, ICA, and lastly the basilar arteries. The mean time of vasospasm development as detected by EEG monitoring and/or TCD was 8.4 ± 2.8 days which was earlier than clinical signs by 12.5 ± 5.3 h in those progressed to DCI.

**Conclusion:**

Continuous EEG monitoring and TCD are valuable methods for early detection of vasospasm and they allow for early therapeutic intervention before irreversible ischemic neurological deficits take place.

## Introduction

Cerebral vasospasm (CV) is one of the leading causes of poorer outcome and higher global disease burden following aneurysmal subarachnoid hemorrhage (aSAH) [1]**.** Cerebral vasospasm is defined as narrowing of a cerebral blood vessel enough to cause reduction in distal blood flow [2]**.** Seventy percent of aSAH patients develop angiographic vasospasm but only 30% progress to develop evident neurological deficits [3]**.** The clinical syndrome occurring because of cerebral vasospasm is called delayed cerebral ischemia (DCI) which is defined as the development of new focal neurological signs and/or as a deterioration in the level of consciousness, lasting for more than 1 h in patients with aSAH [4]**.**

Early detection of DCI is an important step in the way of the improvement of the outcome and the survival of aSAH patients. Transcranial duplex (TCD) is a non-invasive modality which can assess the cerebral blood vessels diameters and flow velocities that can be a useful maneuver in early detection of vasospasm after aSAH [5]**.** Electroencephalogram (EEG) is another sensitive early detector of disturbed neuronal activity due to the reduced cerebral blood flow evidenced by focal slowing of the background activities [6].

## Aim of the work

The study aimed to assess the values of TCD and EEG monitoring in early diagnosis vasospasm following aSAH and the role of early intervention in avoiding the consequences of delayed cerebral ischemia.

## Patients and methods

The first phase of this work was a prospective study conducted on 41 consecutive aSAH patients attending the neurovascular unit and ICUs of the Neurology Department and the Center of Neurology and Psychiatry, Tanta University Hospitals in a 1-year period started in the 1st of May 2015. Thirty-eight patients continued in the study and 3 were excluded because of their clinical deterioration within the first 3 days of onset and their need of artificial ventilation.

Fourteen of the included patients developed cerebral vasospasm (group I) while the remaining 24 patients did not develop vasospasm (group II). Group I was further divided to group Ia consisted of six patients developed transient vasospasm without clinical evidence of DCI and group Ib composed of eight patients developed vasospasm with focal signs of DCI. The latter group underwent interventional intra-arterial nimodipine and/or balloon dilatation of the stenosed segment then it was followed up for 1 week using the National Institute of Health Stroke Scale (NIHSS).

Exclusion criteria included patients with closed temporal window obscuring the TCD assessment and patients with grades 4 and 5 in Hunt and Hess scale for ethical purposes to avoid interruption of their management by the time-consuming EEG monitoring and/or TCD and to avoid the misleading slowing effect of coma on the background EEG activities.

Patients were submitted to aSAH management protocol, Neurology Department, Tanta University Hospital in which SAH was clinically diagnosed on admission and by non-contrast brain CT scan, then the patients were submitted to early CT angiography within 24 h of the onset followed by urgent digital subtraction angiography of cerebral blood vessels for diagnosis of the ruptured aneurysmal site, size, shape, and number. Early interventional embolization of the aneurysms was then done either by simple or balloon assisted coiling.

The study protocol was approved by the local ethics committee. Participation was voluntary and all participants or their first-degree relatives received detailed information concerning the aims of this research work and an informed consent was obtained prior to the commencement of the study.

Subarachnoid hemorrhage was graded clinically using the Hunt and Hess Scale (HHS) and radiologically using the Modified Fisher Scale (MFS). Serial clinical neurological examinations of the patients were done every day using the NIHSS for early detection of new focal neurological signs of DCI.

Transcranial duplex studies were done twice daily, and they started within the 1st 72 h of the onset till the 20th day or the detection of vasospasm using phased array transducer of multi-frequency 1–3 MHz, trans-axial mesencephalic view through the temporal window (Ultrasound Philips. Model: HD 11™ XE, Germany). Vasospasm was diagnosed through measuring the mean flow velocities (MFV) of each of middle, anterior, and posterior cerebral arteries (MCA, ACA, and PCA respectively) and the Lindegaard ratio (LR) (the ratio of MCA velocity to ipsilateral extra cranial ICA velocity) bilaterally. Vasospasm is considered when the MFV of the MCA, ACA, or PCA rises above 120, 90, or 60 cm/s respectively, the MFV of the MCA increases more than 50 cm/s over the first TCD assessment values, or if the LR is more than 3 [7, 8].

EEG recording was done for 1 h three times daily; it started within the first 48 h after admission until the 20th day or the detection of vasospasm signs in the form of polymorphic focal slowing at which the EEG monitoring continued without interruption till recovery or appearance of focal clinical neurological signs at which intervention was done. EEG data were digitalized at a sampling rate of 256 Hz with a high pass filter of 0.08 Hz and a low pass filter of 86 Hz by using Compu-medics, Neuvo 64-512 Channel LTM EEG, Germany (Grael HD EEG Devices –56 channels).

The neuro-interventional machine used in early aneurysmal coiling and vasospasm management was Philips Allura Xper FD20/20 Biplane neuro X-ray System, Netherlands.

Statistical analysis was conducted using SPSS version 19 (Statistical Package for Social Studies) created by IBM, Illinois, Chicago, USA. For numerical values, the range and mean ± SD were calculated. For categorical variables, the number and percentage were calculated and differences between subcategories were tested by Fisher or Monte Carlo exact test as appropriate. *P* value < 0.05 was considered statistically significant.

## Results

The study included 38 aSAH patients aged 50.6 ± 9.8 years, 23 (60.5%) females, 15 (39.5%) males, 18 (47.4%) hypertensive, 7 (18.4%) diabetic, 10 (26.3%) smokers, 1 (2.6%) with cholesterol level below 140 mg/dl, 4 (10.5%) with cholesterol level above 200 mg/dl, 7 (18.4%) overweight with body mass index ≥ 25 kg/m^2^, and 4 (10.5%) chronic daily aspirin users. On admission, the HHS was 1–3 (1.92 ± 0.5), NIHSS was 2–4 (2.3 ± 1.4), and MFS was 1–4 (2.7 ± 0.96).

Angiography showed that the ruptured aneurysmal sites were the anterior communicating (A.com), MCA, posterior communicating (P.com), terminal internal carotid (ICA), and ACA aneurysms in a rate of 23.7%, 36.8%,21.1%, 10.5, and 7.9% respectively. There were no significant differences between groups I and II regarding the sites of ruptured aneurysms (Table 1).

**Table 1:** Demographic data, aneurysmal sites and sizes among patients developed cerebral vasospasm (group I), and those did not develop vasospasm (group II)

		Group I (*n* = 14)	Group II (*n* = 24)	χ^2^	*p*
Sex	Females	8 (57.1%)	15 (62.5%)	0.106	0.74
	Males	6 (42.9%)	9 (37.5%)		
Hypertension		8 (57.1%)	10 (41.7%)	0.84	0.35
Diabetes		3 (21.4%)	4 (16.7%)	0.13	0.71
Smoking		5 (35.7%)	5 (20.8%)	1	0.31
Aneurysmal sites	Anterior communicating	4 (28.6%)	5 (20.8%)	1.18	0.88
	Middle cerebral	6 (42.9%)	8 (33.3%)		
	Internal carotid	1 (7.1%)	3 (12.5%)		
	Posterior communicating	2 (14.3%)	6 (25%)		
	Anterior cerebral	1 (7.1%)	2 (8.3%)		
Aneurysmal size	≤ 12 mm	6 (42.9%)	20 (83.3%)	7.22	0.02
	13–25 mm	7 (50%)	4 (16.7%)		
	> 25 mm	1 (7.1%)	–		

Regarding the aneurysmal size, 26 (68.4%) patients had aneurysms ≤ 12 mm, 11 (29%) had 13–25 mm, and the last 1 (2.6%) had giant aneurysm > 25 mm. Six patients had aneurysms ≤ 12 mm, 7 had aneurysms 13–25 mm, and the included case with giant aneurysm developed vasospasms (Table 1).

The study showed that 14 aSAH patients developed EEG and/or TCD signs of vasospasm; 7 MCA, 3 ACA, 3 supraclinoid ICA, and 1 basilar vasospasm. Patients who developed vasospasm were eight (57.1%) females, six (42.9%) males, eight (57.1%) hypertensive, three (21.4%) diabetic, five (35.7%) smoker, and all these parameters were not significantly different from those did not develop vasospasm (Table 1). Patients who developed vasospasm (group I) were significantly younger and had higher HHS and MFS than group II (41.2 ± 6.2 years, 2.2 ± 0.4 and 2.1 ± 0.86 versus 56 ± 6.9 years, 1.8 ± 0.4 and 2.7 ± 0.96 with *p* ˂ 0.0001, 0.0052, and 0.0031 respectively). There was no significant increase in the time elapsed between symptoms onset and aneurysmal coiling in groups I compared to group II (21.1 ± 6.8 versus 19.8 ± 7.4 with *p* value > 0.05).

The study showed that 35% of included females and 43% of males developed vasospasm. At the same time, 44% of studied A.com aneurysms, 43% of MCA aneurysms, 25% of included ICA, 22% of P.com, and 33% of ACA aneurysms developed vasospasm. The results also showed that 23% of aneurysms ≤ 12 mm, 64% of aneurysms 13–25 mm, and 100% of aneurysms > 25 mm developed vasospasm (Table 2).

**Table 2:** The rate of development of vasospasm regarding patients’ sex, aneurysmal sites, and sizes

		Total (*n* = 38)	Vasospasm (*n* = 14)	No vasospasm (*n* = 24)
			*n* (% of total)	*n* (% of total)
Sex	Females	23 (100%)	8 (34.8%)	15 (65.2%)
	Males	15 (100%)	6 (40%)	9 (60%)
Aneurysmal sites	Anterior communicating	9 (100%)	4 (44.4%)	5 (55.6%)
	Middle cerebral	14 (100%)	6 (42.9%)	8 (57.1%)
	Internal carotid	4 (100%)	1 (25%)	3 (75%)
	Posterior communicating	9 (100%)	2 (22.2%)	7 (77.8%)
	Anterior cerebral	3 (100%)	1 (33.3%)	2 (66.7%)
Aneurysmal size	≤ 12 mm	26 (100%)	6 (23.1%)	20 (76.9%)
	13–25 mm	11 (100%)	7 (63.6%)	4 (36.4%)
	> 25 mm	1 (100%)	1 (100%)	–

The mean onsets of EEG and/or TCD changes related to vasospasm were 8.5 ± 2.9 and 8.5 ± 2.7 days respectively which were non-significantly different. In group Ib, the EEG and TCD changes related to vasospasm preceded the onset of clinically evident DCI by 12.5 ± 5.3 and 12.6 ± 5.6 h respectively. Both EEG and/or TCD signs of vasospasm were earlier in group Ib compared to group Ia (6.95 ± 0.43 versus 10.9 ± 1.12 days with *p* value < 0.001).

The EEG changes in group Ia were non-persistent (3.1 ± 0.6 h) compared to those developed in group Ib which persisted > 5 h till the development of clinically evident DCI. During the EEG changes, the rate of focal background slowing was non-significantly lower in group Ib than group Ia (*p* value > 0.05). At the same time, group Ia patients had significantly slower MCA mean flow velocity and lower LR compared to those who underwent DCI progression (126.4 ± 5.4 and 3.9 ± 0.5 versus 158.4 ± 4.7 and 5.4 ± 0.53 with *p* values < 0.001 and = 0.02 respectively).

**Fig. 1:**
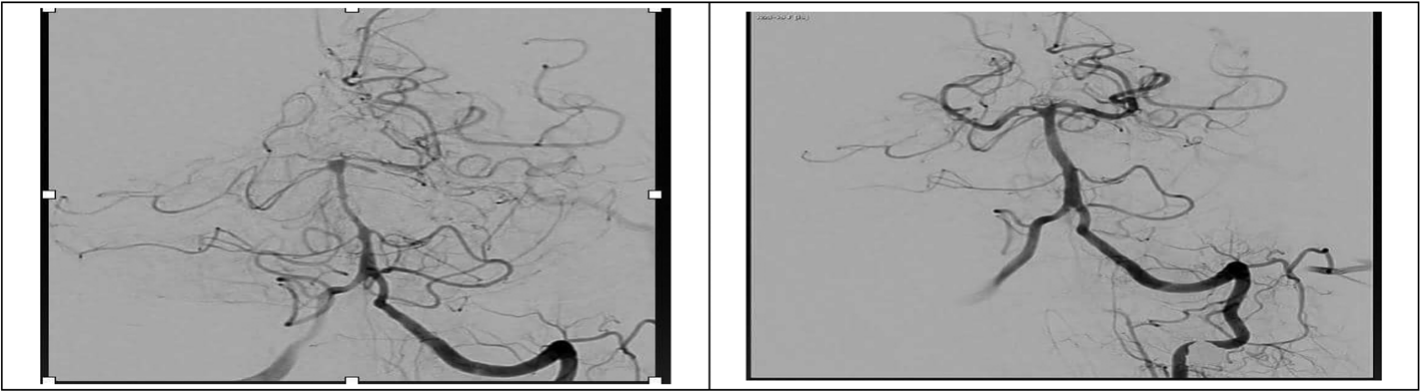
Patient with left MCA vasospasm (left) undergo intervention and improvement of the MCA blood flow after intra-arterial nimodipine injection (right)

**Fig. 2:**
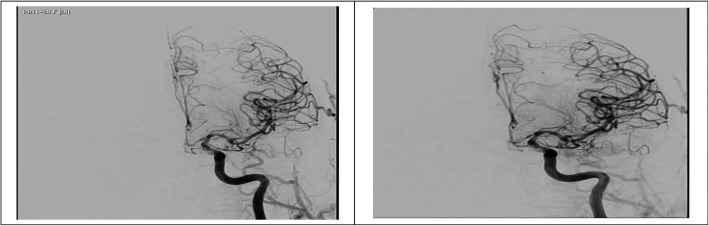
Patient with basilar artery vasospasm (left) undergo interventional intra-arterial nimodipine injection with good improvement of the vasospasm (right)

All members of group Ib patients underwent urgent intervention with intra-arterial injection of nimodipine and/or balloon dilatation of the vasospastic segment (Figs. 1 and 2). All patients showed immediate relief of the stenosed vasospastic segment in angiographic level with the return of normal arterial blood flow. Six patients showed persistent significant improvement of the neurological deficit with NIHSS 14.6 ± 5.6 before intervention and 3.3 ± 1.2 1 day after the procedure. The other two patients (one with MCA and other with ICA vasospasm) developed recurrent vasospasm which needed repeating the intra-arterial nimodipine injection combined with balloon angioplasty. Both patients showed partial recovery of the neurological deficit with NIHSS 18 and 20 before intervention, 14 and 15 1 day after intervention, and lastly 11 and 13 1 week later respectively. The intervention in both patients was 4.3 and 5.1 h after the onset of clinical manifestations which was more delayed than the other six patients (1.8 ± 0.46 h).

## Discussion

Delayed cerebral ischemia is one of the major complications of aSAH and one of the leading causes of high morbidity and mortality. Early diagnosis and management of vasospasm following aSAH is an important step to improve the prognosis [9]**.** The aim of this work was to assess the value of EEG monitoring and TCD as early biomarkers of vasospasm following aSAH and the value of early intervention to avoid the consequences of DCI.

The study showed that ruptured anterior circulation aneurysms were more common than posterior circulation ones, and most of SAH ruptured aneurysms sized ≤ 12 mm. These results passed with the work of Lindner and colleagues [10] who stated that anterior aneurysms are more liable to rupture than posterior ones and they attributed this to genetic factors which result in different anterior aneurysms wall structure compared to posterior ones.

The study also showed that younger patients’ ages are associated with higher risk of developing vasospasm following aSAH. This observation is in accordance with the work of Kale and colleagues [11] and with that of Malinova and colleagues [12] who concluded that younger age < 38 years comes with a risk of development of vasospasm after aSAH, and they attributed this result to the age-related biological factors influencing arterial narrowing and cerebral ischemia. Moreover, they recommended aggressive triple H-therapy in this group of patients immediately after the sealing of the aneurysm.

The study declared that vasospasm following aSAH is more common among patients with higher HHS and MFS which reflects higher amount of blood in the subarachnoid space. These results pass with the work of Burkhardt and colleagues [13] and with that of Zheng and Wong [14] who concluded that more bleeding in the subarachnoid space is associated with higher release of pro-inflammatory mediators including IL-1b, IL-6, and TNFa. These inflammatory mediators result in disruption of blood-brain barrier, cortical spreading depolarization, microvascular spasm and arteriolar constriction, thrombosis with subsequent vasospasm, and increased neuronal response to ischemia.

The study showed that not all patients who developed vasospasm progressed to DCI, but mild cases regressed spontaneously. Patients with severe vasospasm evidenced by prolonged focal EEG slowing and severe TCD abnormalities were more likely to develop DCI. These data had modified the management protocols of aSAH in our institute to allow for intervention when EEG and TCD changes reach levels detecting DCI to avoid the possibility of irreversible neuronal damage. These results are in accordance with the work of Aldakkan and colleagues [15] and with that of Mortimer and colleagues [16] who specified that severe angiographic vasospasm is associated with increased risk of DCI and poorer functional outcome following aSAH.

The study stated that no recorded cases with vasospasm or DCI occurred after the 14th day and the incidence peaked 7th–10th days of symptoms onset. At the same time, patients who progressed to DCI had earlier EEG and/or TCD onset of vasospasm manifestations than those passed a regressive course. This result is in accordance with Aldakkan and colleagues [15] as well as Phan and colleagues [17] who stated that earlier ultrasonographic signs of vasospasm are associated with higher incidence of clinically evident DCI, and they recommend early intervention and aggressive therapy in this sector of patients.

The study declared that EEG monitoring is a reliable non-invasive investigation early able to detect vasospasm following aSAH and predict the possible progression to DCI. This maneuver can save precious several hours which allow for early interventional relief of the vasospasm with consequent better prognosis. Gollwitzer and colleagues [18] as well as Al-Mufti and colleagues [19] agreed with these results and stated that focal reduction in alpha EEG power and decreased alpha/delta ratio could represent valid, observer-independent and non-invasive markers for early detection of vasospasm/DCI following aSAH.

The study also stated that TCD is another early detector of vasospasm and predictor of temporal DCI progression which can save golden hours and allow for early interventional spasmolytic injection to relieve the vasospasm. Mortimer and colleagues [16] as well as Jabbarli and colleagues [20] agreed with and declared these results.

On comparing EEG monitoring with TCD, the study showed that the EEG changes become only valuable after 5 h from their onsets which may consume precious time of these golden hours. At the same time, TCD is more easily applicable when done by a well-trained operator giving it some sort of superiority over EEG monitoring as early detector of vasospasm following aSAH. On the other hand, EEG monitoring is important in those patients with closed temporal TCD window or in absence of well-trained operator.

The study also showed that early intervention by intra-arterial nimodipine injection with or without angioplasty is associated with a good prognosis and a better long-term sequelae in DCI patients after aSAH. These results are agreed by Koenig [21] as well as Patel and colleagues [22] who stated that intra-arterial nimodipine and angioplasty are safe and effective methods for management of vasospasm/DCI after aSAH.

## Conclusion

Continuous EEG monitoring and/or TCD are valuable methods for early detection of vasospasm following aSAH that save precious time and allow for early therapeutic interventional intra-arterial nimodipine with or without angioplasty before irreversible ischemic neurological deficits take place.

## Limitations

The small number of the studied patients with multiple subgrouping sometimes handicapped the statistical analysis but we wish to overcome this point in the second phase of the study.

## Abbreviations

A.com: Anterior communicating artery
ACA: Anterior cerebral artery
aSAH: Aneurysmal subarachnoid hemorrhage
CV: Cerebral vasospasm
DCI: Delayed cerebral ischemia
EEG: Electroencephalogram
HHS: Hunt and Hess Scale
ICA: Internal carotid artery
LR: Lindegaard ratio
MCA: Middle cerebral artery
MFS: Modified Fisher Scale
MFV: Mean flow velocity
NIHSS: National Institute of Health Stroke Scale
P.com: Posterior communicating artery
PCA: Posterior cerebral artery
SAH: Subarachnoid hemorrhage
TCD: Transcranial duplex
